# Discrimination of Patients with Varying Degrees of Coronary Artery Stenosis by ECG and PCG Signals Based on Entropy

**DOI:** 10.3390/e23070823

**Published:** 2021-06-28

**Authors:** Huan Zhang, Xinpei Wang, Changchun Liu, Yuanyang Li, Yuanyuan Liu, Yu Jiao, Tongtong Liu, Huiwen Dong, Jikuo Wang

**Affiliations:** 1 School of Control Science and Engineering, Shandong University, Jinan 250061, China; zhanghuan@mail.sdu.edu.cn (H.Z.); changchunliu@sdu.edu.cn (C.L.); liuyy@sdu.edu.cn (Y.L.); 201814513@mail.sdu.edu.cn (Y.J.); tongtongliu@mail.sdu.edu.cn (T.L.); 201700172023@mail.sdu.edu.cn (H.D.); 201720388@mail.sdu.edu.cn (J.W.); 2 Department of Medical Engineering, Shandong Provincial Hospital Affiliated to Shandong First Medical University, Jinan 250021, China; 230209207@seu.edu.cn; 3 School of Instrument Science and Engineering, Southeast University, Nanjing 210096, China

**Keywords:** joint distribution entropy, cross sample entropy, cross fuzzy entropy, coupling analysis, electrocardiogram, phonocardiogram, coronary heart disease

## Abstract

Coronary heart disease (CHD) is the leading cause of cardiovascular death. This study aimed to propose an effective method for mining cardiac mechano-electric coupling information and to evaluate its ability to distinguish patients with varying degrees of coronary artery stenosis (VDCAS). Five minutes of electrocardiogram and phonocardiogram signals was collected synchronously from 191 VDCAS patients to construct heartbeat interval (RRI)–systolic time interval (STI), RRI–diastolic time interval (DTI), HR-corrected QT interval (QTcI)–STI, QTcI–DTI, Tpeak–Tend interval (TpeI)–STI, TpeI–DTI, Tpe/QT interval (Tpe/QTI)–STI, and Tpe/QTI–DTI series. Then, the cross sample entropy (XSampEn), cross fuzzy entropy (XFuzzyEn), joint distribution entropy (JDistEn), magnitude-squared coherence function, cross power spectral density, and mutual information were applied to evaluate the coupling of the series. Subsequently, support vector machine recursive feature elimination and XGBoost were utilized for feature selection and classification, respectively. Results showed that the joint analysis of XSampEn, XFuzzyEn, and JDistEn had the best ability to distinguish patients with VDCAS. The classification accuracy of severe CHD—mild-to-moderate CHD group, severe CHD—chest pain and normal coronary angiography (CPNCA) group, and mild-to-moderate CHD—CPNCA group were 0.8043, 0.7659, and 0.7500, respectively. The study indicates that the joint analysis of XSampEn, XFuzzyEn, and JDistEn can effectively capture the cardiac mechano-electric coupling information of patients with VDCAS, which can provide valuable information for clinicians to diagnose CHD.

## 1. Introduction

Coronary heart disease (CHD), the most common cardiovascular disease, is characterized by inflammation and fatty deposits along the innermost lining of the coronary arteries. Fatty deposits can gradually thicken and expand over time, forming atherosclerosis, causing stenosis of the arterial lumen, or reducing or blocking blood flowing to the heart, resulting in angina pectoris [1]. Coronary angiography is the gold standard for detecting CHD, but it is invasive, expensive, and prone to complications [2]. Machine learning can effectively mine the hidden information in the cardiovascular system, promising to improve the accuracy of noninvasive CHD detection, which can help to predict patients with varying degrees of coronary artery stenosis before coronary angiography [3].

The heart is a complex system whose active or passive changes can affect the occurrence and transmission of cardiac excitability through the inherent pathways of the heart. The conversion effect between myocardial mechanical activity and electrical activity is called mechano-electric feedback, which is also known as mechano-electric coupling in some studies [4]. Mechano-electric feedback and excitation–contraction coupling form a closed loop in which mechanical change of the heart can affect the electrophysiological state of the myocardium and thus regulate its mechanical function [5]. During electrical or mechanical dysfunction in a healthy heart, mechano-electric feedback maintains myocardial electricity stability by providing feedback regulation. Under the pathological state, the system produces unstable conditions. The pathological process that causes the mechanical changes interfere with this regulation, resulting in clinical syndromes that are difficult to explain from an electrophysiological basis alone [6]. Therefore, coupling analysis of cardiac electrical and mechanical characteristics can describe the functional state and changing laws of the cardiovascular system as a whole, which is of great significance for disease detection [7].

Generally, the common methods of coupling information analysis include coherent function [8,9], cross power spectral density (CPSD) [10], mutual information (MI) [11], phase synchronization [12], multiscale cross approximate entropy [13], cross fuzzy measure entropy [14], and joint symbolic dynamics analyses [15]. Peng et al. [16] utilized cross-recursive quantitative analysis to study the RR interval (RRI)–QT interval (QTI) series of electrocardiogram (ECG) signals in patients with acute myocardial ischemia and found that the complexity of the RRI–QTI series was reduced and the coupling between the two series was weakened during acute myocardial ischemia. Oivasse et al. [17] used correlation coefficients to analyze the dynamic coupling between the RRI and Tpeak–Tend interval (TpeI) series of ECG signals in athletes and healthy subjects. Among numerous coupling analysis methods, entropy-based methods have obtained better results. Li et al. [18] proposed joint distribution entropy (JDistEn) to analyze the coupling between RRI of ECG signals and diastolic time interval (DTI) series of radial artery pressure pulse signals in patients with heart failure, and the results proved that JDistEn has an excellent performance in detecting coupling characteristic. Zhao et al. [19] applied multivariate fuzzy measure entropy to analyze the coupling characteristics between RRI series of ECG signals and S1 and S2 amplitude series of phonocardiogram (PCG) signals under different motion states, and the results proved that the multivariate fuzzy measure entropy method has strong statistical stability and discriminant ability. Furthermore, Ji et al. [20] used linear and nonlinear methods to evaluate the coupling characteristics between the RRI series of ECG signals and the systolic time interval (STI) and DTI series of photoplethysmography signals. They concluded that the cross fuzzy entropy (XFuzzyEn) could better evaluate the differences in the coupling characteristics between CHD patients and healthy subjects. However, in clinical practice, it is more necessary to correctly identify patients with varying degrees of coronary artery stenosis and then give targeted treatment regimens [21]. At present, there has been no relevant research on the combination of multiple coupling analysis methods to more effectively mine cardiac mechano-electric coupling information to distinguish patients with varying degrees of coronary artery stenosis, or on the comparison between entropy-based methods and other coupling analysis methods.

**Figure 1:**
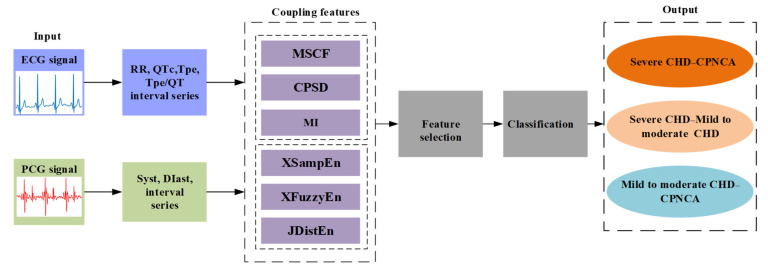
System sketch of multitype coupling features used to distinguish patients with varying degrees of coronary artery stenosis. CPNCA: patients with chest pain with normal coronary angiography.

In this study, six coupling analysis methods were used to extract cardiac mechano-electric coupling features for the identification of patients with varying degrees of coronary artery stenosis. ECG and PCG signals were collected for 5 min synchronously. The RRI, QTc interval (QTcI), TpeI, and Tpe/QT interval (Tpe/QTI) series from ECG signals and the STI and DTI series from PCG signals were extracted to construct the RRI–STI, RRI–DTI, QTcI–STI, QTcI–DTI, TpeI–STI, TpeI–DTI, Tpe/QTI–STI, and Tpe/QTI–DTI series. Subsequently, the cross sample entropy (XSampEn), XFuzzyEn, JDistEn, magnitude-squared coherence function (MSCF), CPSD, and MI were used to capture the coupling information between the series. Then, feature selection was performed using typical support vector machine recursive feature elimination and classification was implemented using excellent XGBoost. The results suggested that the joint analysis of XSampEn, XFuzzyEn, and JDistEn could effectively obtain the cardiac mechano-electric coupling information of patients with varying degrees of coronary artery stenosis. Figure 1 is a system block diagram of multitype coupling features used to distinguish patients with varying degrees of coronary artery stenosis. Our main contributions are as follows.

(1) Patients with varying degrees of coronary artery stenosis are given different treatment programs clinically. At present, other than coronary angiography, there is no effective noninvasive technique for the identification of patients with varying degrees of coronary artery stenosis. Therefore, it is very necessary to accurately identify patients with varying degrees of coronary artery stenosis in clinical practice. In this study, 191 patients with varying degrees of coronary artery stenosis were studied. The classification accuracy for a severe CAD–mild-to-moderate CAD group, severe CAD–chest pain and normal coronary angiography group, and mild-to-moderate CAD–chest pain and normal coronary angiography group was 0.80, 0.77, and 0.75, respectively. The results show that this study can provide a valuable reference for clinicians to diagnose CAD.
(2) Multitype coupling feature sets were constructed. It was verified that the entropy-based coupling feature set was more suitable for the discrimination of patients with varying degrees of coronary artery stenosis.
(3) Dysfunction of the cardiovascular system may result in abnormal electromechanical activity of the heart. In this study, ECG and PCG signals of patients were collected synchronously, and different types of time series intervals related to CAD were extracted. The results confirmed that the coupling series composed of TpeI, Tpe/QTI, DTI, and STI contributed the most to the identification of patients with varying degrees of coronary artery stenosis, which has a guiding significance for the clinical identification of CAD.

## 2. Materials and Methods

### 2.1. Data Acquisition

Data was collected from the Shandong Provincial Qianfoshan Hospital, Jinan, China. There were 191 patients with varying degrees of coronary artery stenosis, including 114 patients with severe CHD, 37 patients with mild-to-moderate CHD, and 40 patients with chest pain and normal coronary angiography. All patients underwent coronary angiography for chest tightness, chest pain, and palpitations. According to the subjects’ self-reports, the symptoms had been present for at least one week at the time of their first visit to the hospital. All data in this study were collected within two days before patients underwent coronary angiography. The inclusion criterion for severe CHD patients was ≥70% stenosis in at least one branch of the left main, left anterior descending, left circumflex, or right coronary artery according to coronary angiography, and those for patients with mild-to-moderate CHD and patients with chest pain and normal coronary angiography were 30~69% and <30% stenosis in the coronary artery branch, respectively. The exclusion criteria were histories of percutaneous coronary intervention, coronary artery bypass surgery, atrial fibrillation, pacemaker rhythm, valvular heart disease, heart failure, and other psychiatric conditions. The basic information of all patients is shown in Table 1. All patients received comprehensive instructions and signed informed consent forms before participating in the data collection. The study was approved by the Institutional Review Board of the Shandong Provincial Qianfoshan Hospital (S374), and it was carried out according to the principles of the Declaration of Helsinki and its amendments.

Before formal collection, patients were required to lie in the supine position for at least 10 min in a quiet and temperature-controlled room (25 ± 3 °C). The ECG and PCG signals of patients were collected simultaneously for 5 min by using a cardiovascular function detection device (CVFD-II, Huiyironggong Technology Co., Ltd., Jinan, China). The CVFD-II instrument includes single lead ECG and PCG signals, extremities air sleeve pressure signals, and a photoplethysmography signals acquisition device. The instrument is mainly used for the evaluation of cardiovascular system function in healthy people and patients with cardiovascular diseases. The ECG signals were collected by the standard lead-I configuration, and the PCG signals were recorded from the third intercostal space at the left border of the sternum. PCG signals were collected by a piezoelectric heart sound sensor. The signal sampling rate was 1 k Hz. The frequency response of ECG signals was 0.5–75 Hz, and the bandpass filter was 0.05–100 Hz. The frequency response of PCG signals was 1–1500 Hz. The PCG sensors were factory-cured to ensure claimed frequency response. During the whole collection, the patients remained quiet and awake to ensure the validity of the signals.

### 2.2. Preprocessing

To get a pure physiological signal, a second-order Butterworth bandpass filter with passband of 0.05–75 Hz and a Butterworth high-pass filter with cutoff frequency of 20 Hz were used to denoise the ECG and PCG signals, respectively. A polynomial third-order Savitzky–Golay filter was applied to eliminate the baseline drift of ECG signals [22]. ECG signal denoising was implemented using the stationary wavelet transform with Symmlet 8 mother wavelet, decomposition of level 4, and a hard thresholding method [23,24]. The IIR Notch filter removed power-line interference at 50 Hz in the ECG and PCG signals.

### 2.3. The Localization of Fiducial Points

#### 2.3.1. ECG Signals

First, the Afonso algorithm [25] was utilized to locate the peaks of the R. The linear phase filter bank decomposes the ECG signal into subbands with uniform frequency bandwidth (5.6 Hz). Due to the subsampling process, the filters are operated once every 89 samples. A variety of features that are proportional to the energy of the QRS complex are obtained by using downsampled signals, and the peak point of R wave is obtained.

For Q-wave detection, two fitting straight lines of the locally normalized ECG signal are obtained by a polynomial of degree 1 in a least-squares sense [26]. Then the two gradients and a smaller intersection angle corresponding to the two fitting lines are computed. The sample with the minimal included angle is regarded as the R peak. Subsequently, respectively before and after the detected R peak, the algorithm researches the onset of the QRS complex according to the same decision strategy

Subsequently, the offset of the T wave was determined by Zhang’s algorithm [27]. The algorithm locates the T wave offsets by calculating an index A (t), which is related to the covered area of the T wave curve. It has been formally proved that the maximum value of A (t) coincides with the T wave offset in each cardiac cycle. The algorithm is robust to noise, waveform morphological change, and baseline drift, and its calculation is simple. One method used for T wave peak detection is to use the trough points from the detection function [28].

#### 2.3.2. PCG Signals

For PCG signals, Springer’s algorithm [29] was used to locate the fiducial points. First, PCG signals were down-sampled to 1 kHz using a polyphase antialiasing filter. Four features of the PCG signals were extracted for segmentation: homomorphic envelogram, Hilbert envelope, wavelet envelope, and power spectral density envelope. Then, the extracted features were normalized and down-sampled to 50 Hz using the above filter. By introducing logistic regression and extended Viterbi algorithm, the segmentation model of the hidden semi-Markov model was modified. Subsequently, the starting and offsets of S1, systolic interval, S2, and diastolic interval were obtained in each cardiac cycle.

**Figure 2:**
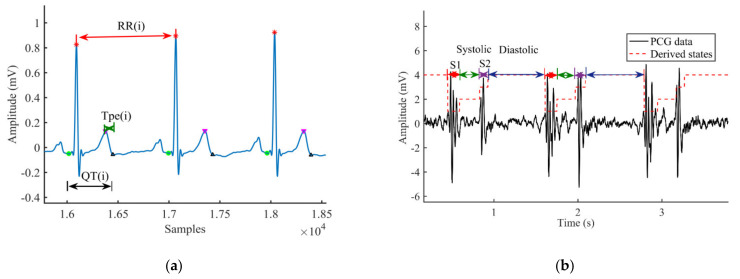
Schematic diagram of time interval extraction of ECG and PCG signals in patients: (**a**) The RRI, QTI, and TpeI series of ECG signals; (**b**) The S1, systolic interval, S2, and diastolic interval of PCG signals.

**Figure 3:**
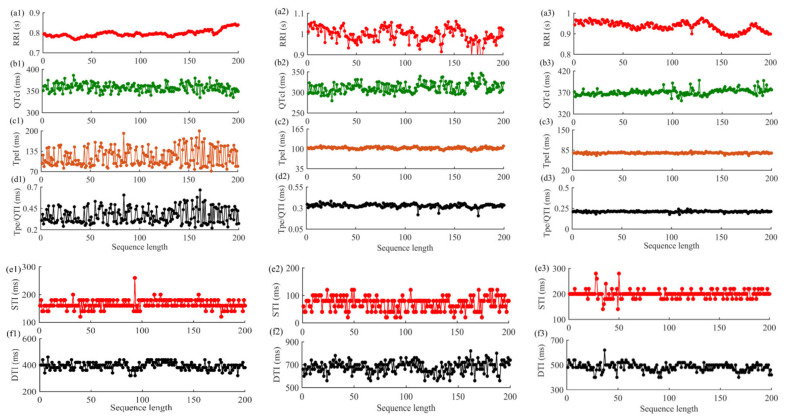
Sample diagram of original interval series of ECG and PCG signals in different types of patients. (**a1**–**f1**) for severe CHD patients; (**a2**–**f2**) for mild-to-moderate CHD patients; (**a3**–**f3**) for patients with chest pain with normal coronary angiography.

**Figure 4:**
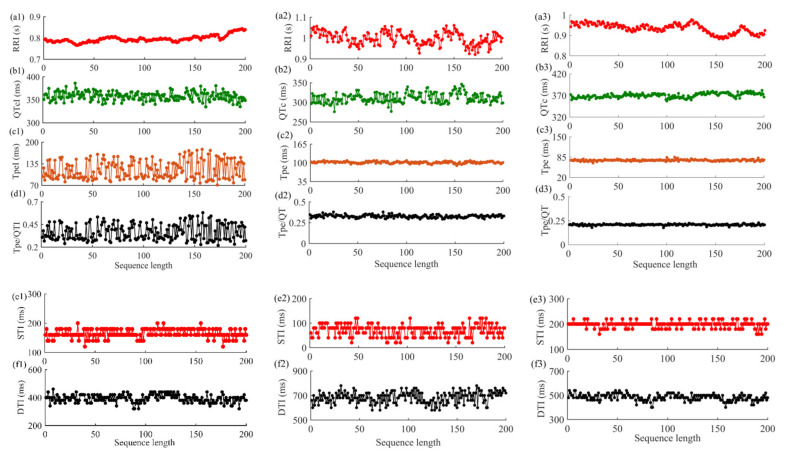
Sample diagram of corrected interval series of ECG and PCG signals in different types of patients: (**a1**–**f1**) for severe CHD patients; (**a2**–**f2**) for mild-to-moderate CHD patients; (**a3**–**f3**) for patients with chest pain with normal coronary angiography.

For single ECG and PCG recordings, the RRI, QTI, TpeI, and Tpe/QTI series were extracted, and QT was corrected by the Bazett method to obtain QTc [30]. The STI and DTI were constructed by using the offsets of S1 and the onsets of S2, the offsets of S2, and the onsets of S1 in the next cardiac cycle, respectively. Subsequently, anomalous intervals were analyzed by the relevant professional. If the percentage of anomalous intervals went beyond 10%, the interval series was considered invalid, and the corresponding subject was excluded from this study [20]. Then, the fiducial points were manually rechecked, and abnormal points were corrected according to relevant literature [29,31,32]. To ensure the effectiveness of signal fiducial point extraction, a few chaotic cardiac cycles (containing uncorrectable fiducial points) in the signal were deleted, and the corresponding cardiac cycles of another signal were also deleted. Figure 2 is the schematic diagram of time interval extraction of ECG and PCG signals in patients. Figure 3 shows an example of the original interval series of ECG and PCG signals in different types of patients. The corresponding corrected time series are shown in Figure 4.

### 2.4. Feature Extraction

#### 2.4.1. XSampEn

Due to the nonlinear and nonstationary characteristics of physiological signals, the entropy analysis algorithm has been widely applied. To better analyze finite series with noise, Richman and Moorman proposed the sample entropy algorithm [33]. To study the cardiac mechano-electric coupling characteristics, we utilized XsampEn as one of the analysis tools, which is defined as:

(1) For the two normalized time series x(i) and y(j), 1<i,j<N, state space reconstruction is carried out to obtain $X_{m}(i)$ and $Y_{m}(j)$, respectively.
  (1) $$X_{m}(i)=[x(i),x(i+1),\ldots ,x(i+m-1)],1\leq i\leq N-m+1$$
  (2) $$Y_{m}(j)=[y(j),y(j+1),\ldots ,y(j+m-1)],1\leq j\leq N-m+1$$
(2) $B^{\left(m\right)}(r)$ is calculated.
  (3) $$d_{i,j}^{\left(m\right)}=\left\|X_{m}(i),Y_{m}(j)\right\|$$
  (4) $$B_{i}^{\left(m\right)}(r)=\frac{1}{N-m}\sum_{j=1}^{N-m}A(r-d_{i,j}^{\left(m\right)})$$
  (5) $$B^{\left(m\right)}(r)=\frac{1}{N-m}\sum_{i=1}^{N-m}B_{i}^{\left(m\right)}(r)$$

Threshold parameter *r* was set as 0.2, and embedding dimension *m* was 2. N is the sequence length. ‖ ‖ is the maximum norm. A(x) is the Heaviside function (i.e., A(x) = 1 if x ≥ 0, otherwise A(x) = 0). Define $B^{\left(m+1\right)}(r)$ similarly according to step (1) and (2).

(3) The XsampEn is defined as follows:
  (6) $$XSampEn(m,r,N)=-\ln(B^{\left(m+1\right)}(r)/B^{\left(m\right)}(r))$$

#### 2.4.2. XfuzzyEn

The threshold parameter *r* is strict, which affects the consistency and statistical stability of the XsampEn algorithm. To overcome this harsh matching process, the Gaussian function (fuzzy membership function) is introduced to replace the Heaviside function, and fuzzy entropy (FuzzyEn) is constructed. Similar to Xsampen, XfuzzyEn can be calculated using fuzzy membership function in step (2) [34].

(7) $$A(d)=e^{-\ln(2){\left(d/r\right)}^{2}}$$

#### 2.4.3. JdistEn

Due to the introduction of the fuzzy membership function, XfuzzyEn avoids the boundary effect of hard threshold discrimination in XsampEn, but it still faces the problem of entropy dependence on the threshold parameter *r*. By measuring the distance matrix globally, the JdistEn avoids the parameter dependence caused by local analysis and solves the problem of entropy dependence on *r* fundamentally [18]. The JdistEn is calculated as follows:

(1) For the normalized time series ${\overset{\wedge }{u}}_{\varphi }(i)$, the state space is reconstructed:
  (8) $$X_{\varphi }(i)=[{\overset{\wedge }{u}}_{\varphi }(i),{\overset{\wedge }{u}}_{\varphi }(i+\tau_{\varphi }),\ldots ,{\overset{\wedge }{u}}_{\varphi }(i+(m_{\varphi }-1)\tau_{\varphi })]$$
  where φ=1,2, i=1,2,…,N−n, $n=\max(m_{\varphi })\times \max(\tau_{\varphi })$, $m_{\varphi }$ is the embedding dimension, and $\tau_{\varphi }$ is the time delay. The input parameters *m* and τ were set at 2 and 3, respectively.
(2) A joint distance matrix is constructed:
  (9) $$JD=J-\sqrt{\left(J-D_{1})(J-D_{2}\right)}$$
  where *J* is the all-ones matrix, and $D_{1}$ and $D_{2}$ are the distance matrix.
(3) Probability density is estimated.

The empirical probability density function (ePDF) of all elements in JD (except the main diagonal) is estimated by the histogram, and the optimal parameter B is obtained by Doane’s formula [35].

(4) The JdistEn is defined as follows:
  (10) $$JDistEn(m,\tau )=-\frac{1}{\log_{2}(B)}\sum_{t=1}^{B}p_{t}\log_{2}(p_{t})$$
  where $p_{t}$ is the probability of each histogram.

For the XsampEn, XfuzzyEn, and JdistEn, we extracted a total of 24 entropy features of 8 interval series between ECG and PCG signals.

#### 2.4.4. MSCF

MSCF can perfectly identify the significant frequency–domain correlation between two series [8]. For two time series x(i) and y(j), 1≤i,j≤N, MSCF is defined as follows:

(11) $$C_{x,y}(f)=\frac{{\left|P_{xy}(f)\right|}^{2}}{P_{xx}(f)P_{yy}(f)}$$

where *N* is the length of the series, and *Pxx* and *Pyy* are the power spectral density estimates of x(i) and y(j), respectively. *Pxy* is the cross power spectral density estimate of x(i) and y(j). The mean and standard deviation of the MSCF of 8 interval series between ECG and PCG signals were extracted in this part, with a total of 16 features.

#### 2.4.5. CPSD

CPSD is a basic tool to measure the similarity between two signals and estimate quantitatively in the form of joint power [10]. For two time series x(i) and y(j), 1≤i,j≤N, CPSD is defined as follows:

(12) $$S_{xy}=\langle XY^{*}\rangle =\langle A_{x}A_{y}\cos(\Delta \phi_{xy})+jA_{x}A_{y}\sin(\Delta \phi_{xy})\rangle$$

where * represents the conjugate complex number; ⟨ ⟩ represents the expectation; X and Y represent the Fourier transform of x(i) and y(j), respectively; $\Delta \phi_{xy}$ represents the phase difference of X and Y at a specific frequency; and $A_{x}$ and $A_{y}$ represent the amplitude of X and Y at a specific frequency, respectively. ICPSD is the absolute value of the imaginary part of CPSD.

(13) $$ICPSD(S_{xy})=\left|imag(S_{xy})\right|=\left|\langle A_{x}A_{y}\sin(\Delta \phi_{xy})\rangle \right|$$

The mean and standard deviation of the ICPSD of 8 interval series between ECG and PCG signals were obtained as the coupling features, and there were 16 features.

#### 2.4.6. MI

MI is an information measure about the correlation between two signals in information theory that has the advantages of simplicity and easy calculation [11]. For two time series x(i) and y(j), 1≤i,j≤N, forming N data pairs (x_i_, y_j_), the MI is defined as [36]:

(1) For given the series *X*, *Y*, N data pairs (*x_i_*, *y_j_*) are formed, *I* = *j* = 1,…, *N*.
(2) For *I* = 1,…,*N*, the probabilities *P_x_*(*x_i_*) and *P_y_*(*y_j_*) are estimated at the sample point using Equations (14)—(17), respectively. *P_x,y_* (*x_i_,y_j_*) is calculated using the same formula:
  (14) $$P_{y}(y_{j})=\frac{1}{n}\sum_{j=1}^{N}K(u),$$
  (15) $$u=\frac{{\left(y-y_{j}\right)}^{T}S^{-1}(y-y_{j})}{h^{2}}$$
  (16) $$K(u)=\frac{1}{{\left(2\pi \right)}^{d/2}h^{d}\det{\left(S\right)}^{1/2}}\exp(-u/2)$$
  (17) $$h={\left\{\frac{4}{\left(d+2\right)}\right\}}^{1/(d+4)}N^{-1/(d+4)}$$
  where *P_x,y_* (*x_i_*,*y_j_*) is the joint probability density of *x* and *y* evaluated at (*x_i_*,*y_j_*), and *P_x_*(*x_i_*) and *P_y_*(*y_j_*) are the marginal probability densities of *x* and *y* evaluated at x_i_ and y_j_, respectively. d was set to 2. *N* is the length of the series. *h* is the kernel bandwidth, and *S*(*y*) is the covariance matrix on the y. *K*(*u*) is the multivariate Gaussian probability density function.
(3) Where the overall dependence between the two series is of interest, one can define the average mutual information ${\bar{I}}_{X,Y}$, as:
  (18) $${\bar{I}}_{X,Y}=\sum_{i,j}P_{x,y}(x_{i},y_{j})\log_{2}\left[\frac{P_{x,y}(x_{i},y_{j})}{P_{x}(x_{i})P_{y}(y_{j})}\right]$$

Eight MI features were obtained from 8 interval series between ECG and PCG signals.

### 2.5. Feature Selection

Support vector machine recursive feature elimination is a sequence backward selection algorithm based on the maximum interval principle of support vector machine, which belongs to the representative wrapper method [37]. It performs a global search on the feature set and eliminates the feature with the least contribution rate in each iteration. After repeated iterations, the model will produce a sort from salient features to nonsalient features to obtain a specified number of optimal feature subsets. Because the support vector machine is adept at processing high-dimensional and small sample data, support vector machine recursive feature elimination has excellent generalization performance. At present, the support vector machine recursive feature elimination method is widely used in feature selection algorithms.

### 2.6. Classification

XGBoost is the representative of ensemble learning algorithms. It is an advanced decision tree gradient enhancement system, and the most important factor for success is its scalability in all scenarios [38]. The boosting algorithm idea is to combine a large number of individually weak but complementary classifiers to generate a strong classifier. The objective function of XGBoost is defined as:

(19) $$L(\phi )=\sum_{i}l(\overset{\wedge }{y_{i}},y_{i})+\sum_{k}\Omega (f_{k})$$

where $l(\overset{\wedge }{y_{i}},y_{i})$ is the differentiable convex loss function that evaluates the difference between the prediction $\overset{\wedge }{y_{i}}$ and the target $y_{i}$, and $\Omega (f_{k})$ penalizes the complexity of the model. Due to the integration of decision trees, XGBoost can solve both classification and regression problems, which has the advantages of fast speed and high accuracy. Meanwhile, XGBoost adopts the second-order Taylor expansion for the objective function, which makes use of the first-order and second-order derivative information of the objective function, so the loss convergence is more accurate. In addition, the regularization term of the objective function helps to smooth the final learning weights to prevent over-fitting. Besides the regularized objective function, XGBoost also uses shrinkage and column subsampling techniques to further prevent overfitting.

## 3. Results

In this study, the data preprocessing and feature extraction codes were run in MATLAB R2018b. Statistical analysis results were obtained by IBM SPSS Statistics (version 26.0, IBM, Armonk, NY, USA), and the machine learning content was performed by Python 3.7. The entire experiment was executed on a PC with 3.30 GHz Intel Core i3 CPU, 6GB of RAM, and a Windows 7 operating system. The results of the statistical analysis, parameter selection, feature selection, and classification among multiple groups are introduced in this section.

### 3.1. Statistical Analysis

**Figure 5:**
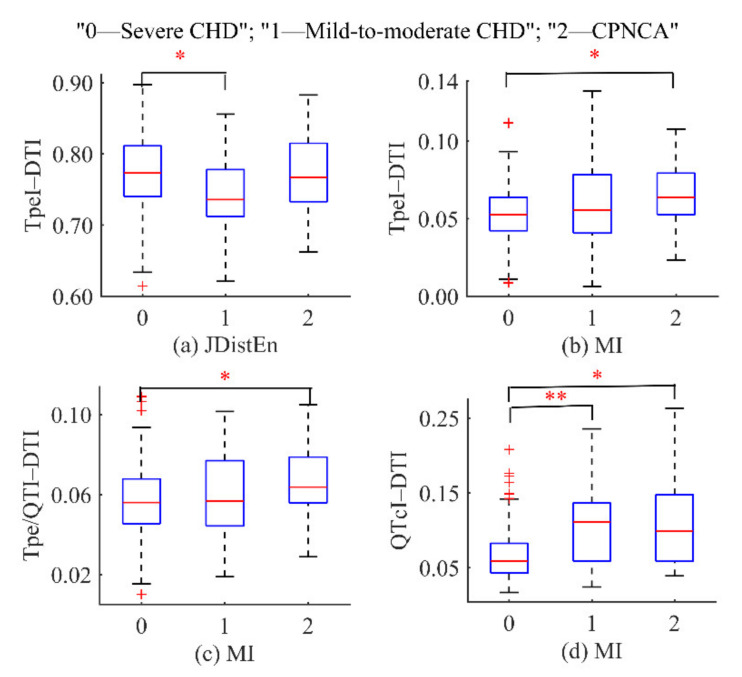
Coupling series with significant differences among different groups: (**a**) for JdistEn method; (**b**–**d**) for MI method. * *p* < 0.05, ** *p* < 0.01.

For coupling feature sets, the normality of the distribution was evaluated by the Kolmogorov–Smirnov test. The one-way ANOVA test approach was used for data with normal distribution, and the post hoc test was adopted for multiple comparisons. Otherwise, the Kruskal–Wallis H test (multiple independent samples) was used. Then, the Bonferroni criterion was employed to correct the multiple comparisons, and the significance level was set at 0.05. The coupling series with significant differences among different groups are shown in Figure 5. It can be seen that the coupling feature of the TpeI–DTI series extracted by the JdistEn algorithm was significantly different in the severe CHD–chest pain and normal coronary angiography group. Then, for the MI algorithm, the coupling features of the TpeI–DTI, Tpe/QTI–DTI, and QtcI–DTI series were significantly different in the severe CHD–chest pain and normal coronary angiography group. The coupling feature of the QtcI–DTI series was significantly different between the severe CHD–mild-to-moderate CHD group.

### 3.2. Parameter Selection

**Figure 6:**
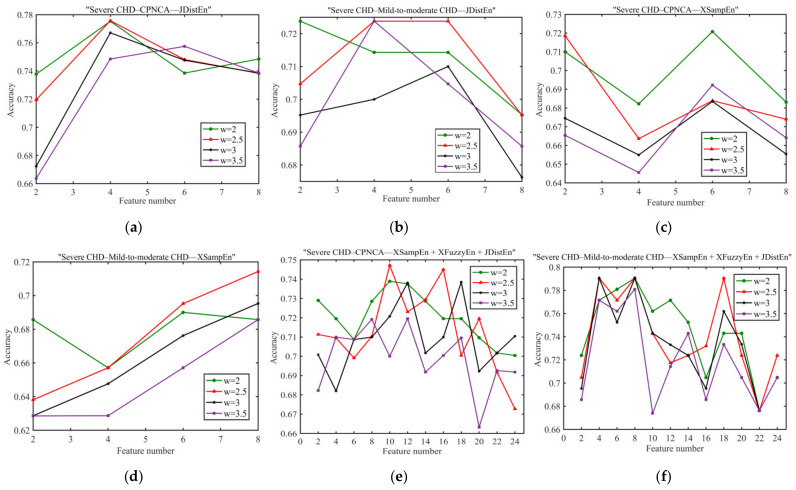
Classification results of different weight coefficients in the optimal method: (**a**,**b**) represent the JDistEn; (**c**,**d**) represent the XSampEn; and (**e**,**f**) represent the joint analysis of XSampEn, XFuzzyEn, and JDistEn.

Since the numbers of patients in the severe CHD group, mild-to-moderate CHD group, and chest pain and normal coronary angiography group were 114, 37, and 40, respectively, the sample proportion was unbalanced, and the analysis results of the severe CHD–chest pain and normal coronary angiography group and the severe CHD–mild-to-moderate CHD group would be affected. Here, an appropriate sample weight coefficient needed to be specified in the XGBoost algorithm to mitigate the impact of sample imbalance on the classification results. The JdistEn and XsampEn algorithms had the best classification accuracy in single algorithm analysis, and the combined analysis of XsampEn, XfuzzyEn, and JdistEn had the best classification accuracy in joint algorithm analysis. Figure 6 illustrates the optimal classification results of different weight coefficients in the verification set for single and joint algorithm analysis. It can be seen that for XsampEn analysis of the severe CHD–chest pain and normal coronary angiography group, the weight coefficient w was set to 2, and the classification accuracy was optimal. In other cases, the classification accuracy was optimal when the weight coefficient w was set as 2.5. Therefore, the weight coefficient w was set as 2.5 in the subsequent analysis.

### 3.3. Feature Selection Results

Table 2 shows the list of optimal feature subsets among different groups. Support vector machine recursive feature elimination was used to select the features of the multitype coupling feature sets based on XSampEn, XFuzzyEn, and JDistEn algorithms, and the optimal feature subsets among different groups were obtained. It can be seen that the optimal feature subsets of different groups all contain the coupling series constructed by RRI, QTcI, TpeI, and Tpe/QTI series in ECG signals and STI and DTI series in PCG signals. Among them, the coupling series constructed by TpeI, Tpe/QTI, DTI, and STI accounted for the main part of the optimal feature subset.

### 3.4. Classification Results

In this study, stratified sampling was conducted on 191 cases of data; 30% (57 cases) of data was extracted as the test set, and the remaining data (134 cases) was used for model training and hyperparameter optimization using fivefold cross validation. Since F1-score was the harmonic average of sensitivity and precision, accuracy and F1-score were used as the main measurement criteria to evaluate the classification performance of the system. The classification results of the single algorithms and the joint algorithms among different groups are presented in Table 3 and Table 4. Results in the two tables were arranged in ascending order according to the classification accuracy and F1-score. It can be seen that, among the single algorithms, JDistEn and XSampEn algorithms had the best classification results. The highest classification accuracies for the severe CHD–mild-to-moderate CHD group, severe CHD–chest pain and normal coronary angiography group, and mild-to-moderate CHD–chest pain and normal coronary angiography group were 0.7826, 0.7021, and 0.7083, respectively. Compared with the joint analysis of MI, CPSD, and MSCF, the joint analysis of XSampEn, XFuzzyEn, and JDistEn revealed the best classification results among the three groups, with the classification accuracy of 0.8043, 0.7659, and 0.7500, respectively, which were all superior to the classification accuracy of the single method.

## 4. Discussion

Cardiac mechano-electric coupling contains important information about the cardiovascular system [5]. Any subtle change should be an important physiological or pathological transformation in clinical, but this is difficult to capture by utilizing traditional methods. As an important measure of chaos theory, entropy has been proved to be able to reveal valuable information hidden in nonlinear complex structures [39]. Entropy is applied to investigate the uncertainty of dynamic system state evolution, which makes the coupling characteristics of the cardiovascular system further studied. For single algorithms, JDistEn and XSampEn were adopted to capture the coupling characteristics between ECG and PCG signals, and the classification results of the three groups were superior to the other four algorithms in this study. For the joint algorithm, the classification results of joint analysis based on XSampEn, XFuzzyEn, and JDistEn were the optimal of the three groups, especially in the mild-to-moderate CHD–chest pain and normal coronary angiography group, where the classification accuracy was 8% higher than that of the joint algorithm based on MSCF, CPSD, and MI. This is consistent with the conclusion of previous studies that entropy-based approaches are better at measuring the nonlinear characteristics of the cardiovascular system [20,33]. This should be because entropy-based methods provide additional information that can be attributed to the idea that inherent coupling in mechano-electric time series may be nonlinear [20]. In addition, compared with the single entropy algorithm, the joint analysis of XSampEn, XFuzzyEn, and JDistEn significantly improved the classification results of the three groups. Because this study focused on cardiac mechano-electric coupling characteristics, the nonlinear features between ECG and PCG signals were extracted, and other multidomain features were not involved, which may be the reason for the relatively weak classification results. The results demonstrated that the joint analysis of XSampEn, XFuzzyEn, and JDistEn can more effectively mine the cardiac mechano-electric coupling information of varying degrees of coronary artery stenosis in patients, which provides valuable information for subsequent classification.

As shown in Figure 4, significant differences were found between the severe CHD and the mild-to-moderate CHD groups. The RRI series was relatively stable in patients with severe CHD. The RRI series fluctuation increased in patients with chest pain and normal coronary angiography, while the series undulation was larger and dispersed in patients with mild-to-moderate CHD. Meanwhile, the DTI series of severe CHD patients and chest pain and normal coronary angiography patients was relatively concentrated, while the series of patients with mild-to-moderate CHD was more floating. Importantly, PCG technology is based on the measurement of variables related to the source (sounds associated with turbulence), not the symptoms, of coronary artery disease. In this important regard it differs from the ECG method, which assesses the cardiac degeneration resulting from an inadequate blood supply [40]. Patients with severe coronary artery stenosis have more obvious changes in their ECG signals. For PCG signals, as the extent of turbulence correlates well with the degree of coronary artery stenosis [41], turbulence can be generated when coronary stenosis is as small as 25%, while turbulence may disappear in occlusion coronary arteries. Hence, PCG technology is more suitable for detecting the early stages of disease [40]. Simultaneously, compared with the other two groups, it can be found that the classification results of both the single algorithm and the joint algorithm in this study were better between the severe CHD and mild-to-moderate CHD groups in Table 3 and Table 4. The results showed that the analysis of cardiac mechano-electric coupling information was more suitable for the division between the severe CHD and mild-to-moderate CHD groups. The joint analysis based on XSampEn, XFuzzyEn, and JDistEn more deeply excavated the cardiac mechano-electric coupling characteristics, which allowed it to more effectively present the differences of characteristics between the two groups.

As shown in Figure 5, MI was used to evaluate the electromechanical coupling characteristics of varying degrees of coronary artery stenosis in patients in different interval series. It was found that the cardiac mechano-electric coupling gradually decreased with the aggravation of the disease, and there was a significant difference between the groups. This is consistent with the conclusion of Ji et al. [20] regarding the reduction of electromechanical coupling in CAD patients. The decrease of time series coupling in CAD patients suggests that cardiovascular system dysfunction may lead to a decrease in the consistency of cardiac electromechanical activity, which may be due to myocardial ischemia induced by CAD. Studies have pointed out that ischemia may cause a decrease in myocardial excitability, conductivity, contractility, and abnormal automaticity, thus affecting the immediate response ability of the cardiac mechanical contractility to electrical excitation [42]. Furthermore, the coupling between QT and RR is known to decrease in situations of sympathetic activation and in patients with a likely increase of the sympathetic drive [43,44]. Healthy aging has also resulted in a decrease in cardiac mechano-electric coupling [45]. Meanwhile, the study of cardiac mechano-electric coupling is also affected by ambient temperature [46]. Therefore, in the data collection stage, we required that the ages of patients in different groups should be matched. Data were collected in a quiet and temperature-controlled room (25 ± 3 °C) to avoid interference factors affecting the analysis results.

The coupling of the cardiovascular system can be evaluated by the interaction between synchronously recorded time series. ECG and PCG signals record the electrical and mechanical activities of the heart during each cardiac cycle and show the functional changes of the cardiac system under different states [7,47,48]. In ECG signals, heart rate variability can reveal the control mechanism of the autonomic nervous system over the heart, and QT interval is a global indicator reflecting ventricular depolarization and repolarization activities [49,50]. They are both common indicators to evaluate the function of the cardiovascular system. Tpe and Tpe/QT have also been proven to be important indicators for predicting cardiovascular events, and they are of great significance for disease prognosis and risk stratification [51,52,53]. Meanwhile, abnormal STI and DTI in PCG signals also play an important role in the evaluation of cardiovascular function [54,55]. The results demonstrated that the RRI, QTcI, TpeI, Tpe/QTI, STI, and DTI series extracted from ECG and PCG signals of patients could be used to distinguish three groups of patients with different types. As can be seen from Figure 4, TpeI and Tpe/QTI series in severe CHD patients were significantly higher than the normal range (40–110 and 0.15–0.25, respectively) [51], with great fluctuations. The TpeI and Tpe/QTI series of patients with mild-to-moderate CHD fluctuated slightly, while the series of patients with chest pain and normal coronary angiography were more concentrated and stable. The statistical analysis results in Figure 5 showed that the coupling features of TpeI, Tpe/QTI, QTcI, and DTI were significantly different in the severe CHD–chest pain and normal coronary angiography group and the severe CHD–mild-to-moderate CHD group. Furthermore, for the optimal feature subset in Table 2, the results were consistent with the above results, and the coupling series constructed by TpeI, Tpe/QTI, DTI, and STI were the main components of different groups. Therefore, we suggest that the coupling series constructed by TpeI, Tpe/QTI, STI, and DTI could be given priority for the differentiation of patients with varying degrees of coronary artery stenosis.

As can be seen from Table 3 and Table 4, both the single algorithm and the joint algorithm had low classification specificity in the severe CHD–chest pain and normal coronary angiography group and the severe CHD–mild-to-moderate CHD group. However, for the mild-to-moderate CHD–chest pain and normal coronary angiography group, the classification specificity of the algorithms was relatively high, and the AUC values were also significantly better than the other two groups. This may be due to sample imbalance [56], as there were 114 patients with severe CHD, 37 patients with mild-to-moderate CHD, and 40 patients with chest pain and normal coronary angiography in this study. The data of this study came from the Department of Cardiology, Shandong Provincial Qianfoshan Hospital. As the majority of subjects in the Department of Cardiology were severe CHD patients, the proportion of different patients in the samples was unbalanced. During the follow-up data collection, we will conduct data matching among groups to reduce the impact of data imbalance on the study.

In addition, the severe CHD patient in Figure 4 also had arrhythmia, and the changes of c1 and d1 series may also be related to the influence of arrhythmia. Studies have shown that the change of QT variability (QTV) may be the combined result of the change of QTV itself and the influence of HRV [57]. Even though Bazzett’s method was used to correct QT in this study, some studies have shown that there is lag in the impact of HRV on QT change [57], which makes the relationship between QT and HR more complicated. In addition, the variability of QT and Tpe series is also influenced by circadian rhythm factors, and the prolonged duration of QT and Tpe interval is more significant in people with hypertension, obesity, and smoking [58]. Meanwhile, compared with Figure 3 and Figure 4, the interval series of ECG and PCG signals were affected by noise and artifacts, so the step of fiducial points correction is particularly important.

There are several limitations in this study. Due to the sample imbalance between groups, the classification specificity was low, which suggests that we need to expand the collection range and increase the collection period to obtain more data for sample balance in the later stage. In addition, there were nonstationary data series in the sample. The presence of nonstationary data sequences in the sample increases the likelihood of overestimating the effect of sympathetic control, possibly affecting the ability of statistical testing [59]. In this study, we only differentiated patients with varying degrees of coronary artery stenosis into two categories among multiple groups instead of multi-classification. In the future, we will further study a more effective method to excavate the mechano-electric coupling characteristics and try to conduct a multi-classification study on patients with varying degrees of coronary artery stenosis.

## 5. Conclusions

In this study, we explored single algorithms and joint analysis algorithms to distinguish three groups of patients with varying degrees of coronary artery stenosis. The RRI–STI, RRI–DTI, QTcI–STI, QTcI–DTI, TpeI–STI, TpeI–DTI, Tpe/QTI–STI, and Tpe/QTI–DTI series from 191 patients with VDCAS were extracted. The XSampEn, XFuzzyEn, JDistEn, MSCF, CPSD, and MI algorithms were used to analyze the coupling characteristics between the series. Then, feature selection was performed by support vector machine recursive feature elimination, and the XGBoost method was used for classification. Finally, the results certified that JDistEn and XSampEn were suitable for capturing the coupling information of patients with varying degrees of coronary artery stenosis. The joint analysis of cross entropy reveals the potential value of the entropy algorithm in the series analysis of cardiac electromechanical activity, which could provide valuable information for clinicians to diagnose CHD.

## Figures and Tables

**Table 1:** Basic information of all patients.

Characteristic	CPNCA Group	Mild-to-Moderate CHD Group	Severe CHD Group
Male/female	20/20	20/17	71/43
Age (year)	60 ± 11	63 ± 7	65 ± 9
Height (cm)	163 ± 7	165 ± 7	166 ± 7
Weight (kg)	69 ± 13	68 ± 9	69 ± 11
Body mass index (kg/m^2^)	26 ± 3	26 ± 3	25 ± 3
Systolic blood pressure (mmHg)	128 ± 16	133 ± 16	137 ± 18
Diastolic blood pressure (mmHg)	81 ± 11	81 ± 9	85 ± 16

Information was expressed as male/female or mean ± standard deviation. CPNCA: chest pain with normal coronary angiography.

**Table 2:** Optimal feature subsets among different groups.

Severe CHD–CPNCA	Severe CHD– Mild-to-Moderate CHD	Mild-to-Moderate CHD–CPNCA
RRI–STI–XS	RRI–STI–XF	RRI–STI–XS
RRI–STI–JD	RRI–STI–JD	RRI–STI–XF
RRI–DTI–JD	RRI–DTI–XF	QTcI–STI–XS
QTcI–STI–XS	RRI–DTI–JD	QTcI–DTI–XF
TpeI–STI–XF	QTcI–STI–XF	QTcI–DTI–JD
TpeI–STI–JD	QTcI–DTI–XF	TpeI–STI–XS
TpeI–DTI–XF	QTcI–DTI–JD	TpeI–DTI–XS
Tpe/QTI–STI–XS	TpeI–STI–XS	TpeI–DTI–JD
Tpe/QTI–STI–XF	TpeI–STI–XF	Tpe/QTI–STI–XS
Tpe/QTI–STI–JD	TpeI–STI–JD	Tpe/QTI–STI–JD
	TpeI–DTI–XS	Tpe/QTI–DTI–XS
	TpeI–DTI–XF	Tpe/QTI–DTI–JD
	TpeI–DTI–JD	
	Tpe/QTI–STI–XF	
	Tpe/QTI–STI–JD	
	Tpe/QTI–DTI–XS	
	Tpe/QTI–DTI–XF	
	Tpe/QTI–DTI–JD	

Note that the RRI–STI–XS form represents the XSampEn feature of RRI–STI series. XS: XSampEn, XF: XFuzzyEn, JD: JDistEn. CPNCA: patients with chest pain with normal coronary angiography.

**Table 3:** Comparison of classification results of single algorithm.

Groups	Methods	Accuracy	F1-Score	Sensitivity	Specificity	AUC
Severe CHD– Mild-to-moderate CHD	MI	0.6522	0.7714	0.7714	0.2727	0.5792
	XFuzzyEn	0.7174	0.8267	0.8857	0.1818	0.5636
	JDistEn	0.7391	0.8286	0.8286	**0.4545**	0.6195
	MSCF	0.7391	0.8286	0.8286	**0.4545**	**0.6468**
	CPSD	0.7608	0.8571	0.9429	0.1818	**0.6415**
	XSampEn	**0.7826**	**0.8718**	**0.9714**	0.1818	0.5156
Severe CHD–CPNCA	XSampEn	0.6383	0.7536	0.7429	**0.3333**	0.5667
	XFuzzyEn	0.6596	0.7714	0.7714	**0.3333**	0.5845
	MSCF	0.6808	0.7945	0.8286	0.2500	0.5976
	CPSD	0.7021	0.8108	0.8571	0.2500	0.4476
	MI	0.7021	0.8000	0.8571	0.1667	**0.6619**
	JDistEn	**0.7021**	**0.8158**	**0.8857**	0.1667	0.4238
Mild-to-moderate CHD–CPNCA	MI	0.5000	0.5714	0.6667	0.3333	0.5000
	XFuzzyEn	0.5833	0.6154	0.6667	0.5000	0.5417
	XSampEn	0.6667	0.6364	0.5833	0.7500	0.7049
	CPSD	0.7083	0.6667	0.5833	**0.8333**	0.7638
	MSCF	0.7083	0.6957	0.6667	0.7500	**0.7743**
	JDistEn	**0.7083**	**0.7200**	**0.7500**	0.6667	0.7222

CPNCA: patients with chest pain with normal coronary angiography. The best performance between different groups is marked with bold.

**Table 4:** Comparison of classification results of the joint algorithm.

Groups	Methods	Accuracy	F1-Score	Sensitivity	Specificity	AUC
Severe CHD– mild-to-moderate CHD	MI–CPSD–MSCF	0.7826	0.8650	0.9143	**0.3636**	**0.6182**
	XSampEn–XFuzzyEn–JDistEn	**0.8043**	**0.8831**	**0.9714**	0.2727	0.6078
Severe CHD–CPNCA	MI–CPSD–MSCF	0.7447	0.8333	0.8571	**0.4167**	**0.6500**
	XSampEn–XFuzzyEn–JDistEn	**0.7659**	**0.8571**	**0.9428**	0.2500	0.5047
Mild-to-moderate CHD–CPNCA	MI–CPSD–MSCF	0.6667	0.6923	0.7500	0.5833	0.5694
	XSampEn–XFuzzyEn–JDistEn	**0.7500**	**0.7000**	0.5833	**0.8299**	**0.8290**

CPNCA: patients with chest pain with normal coronary angiography. The best performance between different groups is marked with bold.

## Data Availability

The data presented in this study are available on reasonable request from the corresponding author, X.W. The data are not publicly available due to their containing information that could compromise the privacy of research participants.
